# Intensive, personalized multimodal rehabilitation in patients with primary or revision total knee arthroplasty: a retrospective cohort study

**DOI:** 10.1186/s13102-020-0157-1

**Published:** 2020-01-10

**Authors:** Jesper Bie Larsen, Lisbeth Mogensen, Lars Arendt-Nielsen, Pascal Madeleine

**Affiliations:** 1 Center for Sensory-Motor Interaction (SMI), Department of Health Science and Technology, School of Medicine, Aalborg University, Fredrik Bajers Vej 7, Bld. D3, DK-9220 Aalborg East, Denmark; 2 Sports Sciences – Performance and Technology, Department of Health Science and Technology, School of Medicine, Aalborg University, Niels Jernes Vej 12, DK-9220 Aalborg East, Denmark; 3 Montebello – Department of rehabilitation, North Zealand’s Hospital, Juan Luis Peralta 30, 29639 Benalmádena, Spain

**Keywords:** Total knee replacement, Revision, Pain, Intensive multimodal rehabilitation, Physical therapy

## Abstract

**Background:**

Recent evidence has shown that many patients suffer from persistent pain and impaired function after primary or revision total knee arthroplasty (TKA). Post-surgical complications may in addition decrease physical performances and lead to more pain and impacted quality of life.

The purpose of the study was to assess the changes in pain intensity and functional capacity among patients with post-surgical complications after TKA three weeks of intensive, personalized multimodal rehabilitation.

**Methods:**

A retrospective cohort study consisting of 217 patient of which 166 had primary TKA and 51 had revision TKA was conducted. On average, primary TKA patients and revision TKA patients were 3.7 and 2.7 months post-surgical, respectively. All patients have had post-surgical complications and were referred to an inpatient rehabilitation department, where they received a personalized three-week intensive, multimodal rehabilitation protocol. The rehabilitation consisted of sessions targeting neuromuscular function, postural control, and flexibility, sessions focusing on improving muscle strength and cardiovascular function and sessions with focus on gait retraining. The frequency of training was 2–4 sessions/day. The primary outcome was the Knee injury and Osteoarthritis Outcome Score (KOOS) and secondary outcomes were pain intensities measured using numerical rating scale, 6 min. walking test, stair-climbing test and range of motion for knee flexion and extension. Outcome measures were assessed at baseline upon referral and at follow-up before discharge.

**Results:**

All outcomes, except pain at rest in the revision group, improved significantly. KOOS subscales, improved 8.5 to 14.2 in the primary TKA group (*p* < 0.001) and 6.9 to 10.8 in the revision group (*p* < 0.001). For the TKA group, effect sizes were medium-to-large for all KOOS subscales, 6 min. walking test, stair-climbing test, and pain intensity during activity. For the revision group, effect sizes were medium-to-large for KOOS subscales symptoms and activity of daily living, 6 min. walking test, stair-climbing test, and knee flexion.

**Conclusion:**

Patients with post-surgical complications after primary or revision TKA experienced clinical relevant improvement in self-reported outcomes, pain relief, and improved physical performances after three weeks of personalized multimodal rehabilitation. The results suggest that an intensive, multimodal approach might be useful to obtain clinically relevant improvements.

## Background

Osteoarthritis (OA) is considered the most frequent cause of disability and pain in the elderly population, and the knee joint is one of the joints most commonly affected [1, 2]. End-stage knee OA is often treated with knee replacement and primary total knee arthroplasty (TKA) is considered an effective treatment for pain relief, improved function and hence quality of life [3, 4]. However, it is well documented that persistent pain and functional disability occur in a large proportion of the patient after an otherwise technically successful TKA [5, 6]. Studies have reported chronic pain rates after primary TKA at 12 months post-operative in 13–17% of the patients and chronic pain rates at 2–7 years post-operative varying between 8 and 27%. Furthermore, 20% of patients 6 months post-operative stated that their primary TKA was not successful in allowing them to resume their regular physical activities [6]. As for patients undergoing revision TKA, a study by Petersen et al. found that 47% of the patients reported severe or unbearable pain 3 years after their latest surgery and regarding functional ability, 37% of the patients were unable to walk a distance > 0.5 km. In general, the patients undergoing revision surgery were less satisfied with their surgery than the patients undergoing primary TKA and revision TKA based solely on the presence of pain cannot be recommended [7, 8].

Pain and surgery lead to a temporary loss of motor function and muscle strength, which often seems to improve in the post-operative phase, especially following a rehabilitation regimen [9–14]. When patients exhibit pain or other post-surgical complications, their rehabilitation may be compromised and result in impaired function, loss of muscle strength and poor postural control. Which rehabilitation strategy is the most optimal has yet to be resolved [15]. It has been proposed that some patients, who does not respond adequately to standard rehabilitation, require a more intensive rehabilitation approach to recover [15–17]. Furthermore, it has also been suggested that rehabilitation should be comprehensive in terms of muscle groups targeted and include progressive exercises [18]. A case study examined patients with post-surgical complications after TKA and found that quadriceps muscle weakness, knee flexion contracture or flexion deficit, muscle tightness and impaired gait were frequently observed in these patients [19]. With an intensive, multimodal treatment approach, the study reduced symptoms and improved function, but the authors did not specify the intervention or used any validated outcome measures, making the validity of the results questionably and treatment recommendations difficult.

Based on the lack of knowledge about management of patients with post-surgical complications after TKA, it is necessary to study the outcomes of intensive multimodal rehabilitation. Hence, there is a need for clinical studies using “best practice” as treatment and including validated outcomes measures to allow for interpretation of the benefits from the treatment. This may elucidate whether intensive rehabilitation can be an effective approach in patients with complications after primary or revision TKA. Therefore, the purpose of this retrospective pragmatic cohort study in real-life settings was to investigate the short-term effect of 3-weeks of intensive, multimodal rehabilitation in patients with complications after primary or revision TKA . The hypothesis was that self-reported outcomes and objective physical performance would improve and pain levels would decrease. Although this observational cohort study did not include a control group, it could provide important information of effect sizes and thereby, be used for designing future randomized controlled trials.

## Methods

### Patient selection

**Fig. 1:**
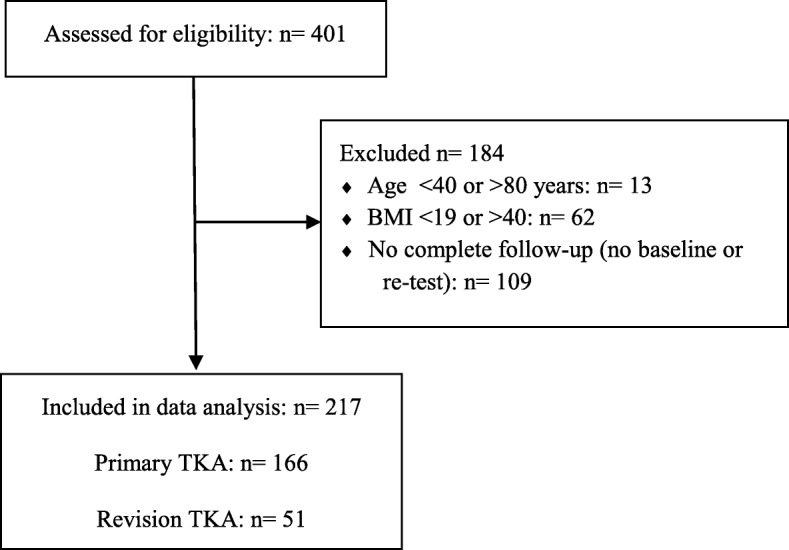
Flow chart. BMI: Body Mass Index. TKA: Total knee arthroplasty

The study was a retrospective register-based study based on data collected from consecutive patients in the period from February 2017 to June 2018. All patients had been hospitalized at Montebello – Department of Rehabilitation, North Zealand Hospital, Denmark. All patients with primary or revision TKA were referred to the rehabilitation department by their surgeon or family physician, due to post-surgical complications. The main reasons for referral was persistent pain and impaired physical function due to lack of effect from initial rehabilitation, infection, loosening of implants and/or revision surgery. Inclusion criteria was 1) patients with primary TKA or revision TKA, 2) 40–80 years old, 3) body mass index (BMI) within 19–40 kg/m^2^ and 4) post-surgical complications based on surgeon or family physician examination. Exclusion criteria were lack of complete follow-up and lack of ability to adhere to the treatment. Overall, 166 patients with primary TKA and 51 patients with revision TKA were included in the analysis (Fig. 1). Since all patients were referred to rehabilitation and therefore, must receive the treatment, it was not possible to sample a control group. The study was conducted in accordance with STROBE guidelines and followed the TIDieR guidelines for reporting interventions (Additional file 1) [20, 21]. Due to the retrospective and the quality control nature of the study, no approval from the local ethics committee of the North Denmark Region was required. The study was approved by the Danish Data Protection Agency.

### Intervention

All patients referred to Montebello – Department of Rehabilitation undergo a standardized, personalized multimodal treatment protocol. The protocol is a 3-week stay in a rehabilitation hospital during which patients receive intensive, multimodal rehabilitation supervised by physiotherapists. The rehabilitation consisted of sessions targeting neuromuscular function, postural control, and flexibility, sessions focusing on improving muscle strength and cardiovascular function and sessions with focus on gait retraining. Rehabilitation was based on evidence-based exercise programs [22, 23] and included exercises such as pelvic lifts, sit-ups, sliding exercises, lunges, rubber band exercises, and functional movements like chair stands and stair climbing. The frequency of training was two to four sessions per day, lasting from 30 to 50 min. pr. session (for further information on the rehabilitation protocol - see Additional file 2). Exercises were personalized, i.e., adjusted to individual level during the group-based rehabilitation based on physical ability, fatigue and pain level. The total magnitude of exercises was also adjusted on individual basis in order to avoid a “one size fits all” approach since patients have different physical capacity and report different pain intensities. Educational sessions with focus on pain management, information on their prosthesis, relevance of exercise as treatment and how to continue exercising on their own after discharge were provided including teaching self-mobilization techniques to improve knee range of motion (ROM). Patients were gathered in groups of 11 with the same area of problems (i.e. knee) and the same physiotherapist supervised the group for the entire intervention period.

### Assessment

All patients referred to the hospital undergoes a test battery at baseline after admission and at re-test before discharge, measuring self-reported outcomes, pain intensity, physical performance during activities of daily living (ADL) and active knee ROM, which are areas that has previously been highlighted as important quality indicators for rehabilitation in clinical practice after TKA [24, 25]. In the present study, the Knee injury and Osteoarthritis Outcome Score (KOOS) was used as the primary outcome. KOOS is a subjective questionnaire covering five subscales of pain, symptoms, activities of daily living (ADL), sports/recreation and quality of life (QOL). Each KOOS subscale consists of multiple items, scored on a 5-point Likert scale; The KOOS ranges from 0 (worst) to 100 (best) [26]. KOOS is a patient self-reported outcome measure reported to be valid and reliable during short-term and long-term follow-up in patients with TKA [27]. An 8–10 point improvement is commonly used as a minimal clinically important difference (MCID) [28]. In the present study, the mean change from baseline to 3-weeks follow-up of 4 subscales evaluating pain, symptoms, activities of daily living and quality of life was used as primary outcomes. The subscale sport/recreation was not included since it measures strenuous activities, which this patient cohort is not expected to participate in and often is contraindicated by the surgeon. This approach was in accordance a previous study within the field of osteoarthritis [29].

Secondary outcomes consists of pain intensity ratings and physical performance measurements. For the index knee, the pain intensity during rest and during activity (i.e. walking, stair climbing) was assessed. A numerical rating scale (NRS) was used, where “0” represents “no pain” and “10” represents “maximal pain”.

The included physical performance measures are part the recommended core set of outcome measures from the OsteoArthritis Research Society International (OARSI) [30]. The 6 min. walk test evaluates the ability to walk a longer distance and the aerobic capacity. The participants were asked to walk as quickly and as safely as possible, without running, along a 21 m. walkway and then turn around a cone, return, and then repeat to cover as much ground as possible on 6 min. Regular walking aid was allowed and as well as resting periods, if necessary. If safety was of concern, the tester followed slightly behind and off to one side of the participant, but not as to pace or impede the participant. The amount of meters covered in 6 min. was the outcome score [30]. The stair climb test consisted of ascending and descending stair activity and thereby was a test of lower body strength and balance. A staircase with 22 stairs was climbed up and down once and participants were asked to ascend and descend stairs as quickly and as safely as possible. Use of a handrail and walking aid were permitted if needed. The amount of time it took to complete the ascending and descending of the stairs was the outcome score [30]. Active knee ROM was assessed in the affected knee. For knee extension, the patient was lying supine and the knee was resting on the examination couch. The axis of the goniometer was aligned on the lateral aspect of the knee joint with one arm of the goniometer in line with the femur and the other in line with the tibia. Keeping the goniometer in place, the knee was actively extended as fully as possible while recording the angle in degrees. The largest angle of the three measurements was noted. For knee flexion, the patient was lying supine and the knee resting on the examination couch. The axis of the goniometer was aligned on the lateral aspect of the knee joint with one arm of the goniometer in line with the femur and the other in line with the tibia. Keeping the goniometer in place and the clinician supporting the weight of the limb, the knee was actively flexed fully with recording of the angles in degrees. The highest of three measurements was recorded [31]. Furthermore, demographic variables such as sex, age, BMI, index leg side, time-since-surgery and reasons for revision surgery were collected.

## Data analysis

Data was checked for normality by assessing data frequency in histograms, QQ-plots, and Shapiro-Wilk tests.

For normal distributed outcomes, paired samples t-tests for all continuous outcomes were applied. Data is presented as mean (SD) unless otherwise stated. For the non-normal distributed outcomes, Wilcoxon signed rank test were used and data is presented with median and min-max.

Effect sizes were calculated as Cohen’s *d* = (*Mean*_After_ - *Mean*_Before_)/*SD*_pooled_. Effect sizes were interpreted as 0.2 = “small” effect size, 0.5 = “medium” effect size and 0.8 = “large” effect size, as suggested by Cohen [32].

The significance level was set to 0.05 and results are displayed with 95% confidence intervals. All analysis were performed with the use of the statistical software SPSS, Version 25.0 (SPSS Inc., Chicago, IL, USA).

## Results

### Demographics

A total of 217 patients (primary TKA: 166, Revision TKA: 51) were included in the analysis. The mean age in each group was 62 and 64 years, respectively. The mean time-since-surgery was 3.7 and 2.7 months in each group (Table 1). All patients with primary TKA had surgery due to end-stage knee OA. Patients with revision TKA had surgery because of infection (14%), implant loosening or instability (51%), pain (20%) or other reasons (15%). A proportion of patients (27%) were excluded from the analysis due to lack of complete data at both baseline or re-test. The major reasons behind the missing data were patient illnesses or early discharges as well as no data available from the KOOS questionnaire, due to a change in outcome measures in the study period. Baseline characteristics of excluded patients can be seen in Table 1. There no significant differences between the characteristics of the included and excluded patients. All included patients concluded three weeks of intensive, multimodal rehabilitation and no side effects were observed.

**Table 1:** Patient characteristics for patients included and for those excluded. Values are mean (SD) unless otherwise stated

	Included patients		Excluded patients	
	Primary TKA (n: 166)	Revision TKA (n: 51)	Primary TKA (n: 68)	Revision TKA (n: 23)
Age (years)	64.0 (8.6)	62.2 (8.8)	64.2 (8.7)	61.7 (9.8)
BMI (kg/m^2^)	28.9 (4.3)	28.3 (5.0)	28.7 (4.5)	29.4 (5.2)
Sex (females, %)	114 (69%)	30 (59%)	48 (71%)	14 (61%)
Index leg (right, %)	87 (52%)	30 (59%)	29 (43%)	12 (52%)
Time-since-surgery (months)^a^	3.7 (5.9)	2.7 (2.4)	2.9 (3.0)	2.0 (1.2)

*TKA* Total Knee Arthroplasty. *BMI* Body Mass Index. ^a^ Time-since-surgery is the period from the date of surgery (TKA or revision TKA) to the date of starting the rehabilitation

### Primary outcome - KOOS

Both the primary TKA and the revision TKA group had significant improvement of all 4 subscales in KOOS. For the primary TKA group there was a mean improvement in the subscales, ranging from 8.5 to 14.2. For the revision TKA group, there was a mean improvement in the subscales, ranging from 6.9 to 10.8. Both groups had the largest improvement in the ADL subscale (Table 2). Effect sizes were 0.57 for KOOS Pain, 0.58 for KOOS Symptoms, 0.88 for KOOS ADL and 0.51 for KOOS QOL for the primary TKA group. For the revision TKA group, effect sizes were 0.38 for KOOS Pain, 0.54 for KOOS Symptoms, 0.70 for KOOS ADL and 0.46 for KOOS QOL.

**Table 2:** The Knee injury and Osteoarthritis Outcome Score subscale. Values are mean (SD)

	Primary TKA (n: 166)				Revision TKA (n: 51)			
	Before	After	Change	95% CI	Before	After	Change	95% CI
KOOS Pain	54.1 (17.4)	63.9 (17.8)	9.8* (14.2)	7.6; 12.0	52.3 (17.2)	59.3 (19.3)	6.9* (17.0)	2.2; 11.7
KOOS Symptoms	52.8 (16.3)	62.1 (15.3)	9.4* (13.7)	7.3; 11.5	52.4 (15.5)	60.6 (15.0)	8.2* (14.9)	4.0; 12.4
KOOS ADL	58.0 (15.8)	72.2 (15.5)	14.2* (13.2)	12.2; 16.2	58.4 (15.6)	69.1 (15.1)	10.8* (14.0)	6.8; 14.7
KOOS QOL	39.2 (18.0)	47.7 (17.4)	8.5* (15.6)	6.1; 10.9	36.5 (18.1)	45.1 (18.9)	8.6* (18.3)	3.5; 13.8

*ADL* Activities of daily living, *QOL* Quality of life, *: *P*-value < 0.05.

### Secondary outcomes – pain, physical performances and active knee ROM

For the primary TKA group, significant decreases in NRS pain intensity at rest and during activity were reported and a mean decrease on NRS of 1.7 were observed during activity. For the revision TKA group, a significant pain reduction on NRS of 0.8 was reported during activity. At rest, there was a tendency to lower pain intensity on NRS, although non-significant (Table 3). Effect sizes were 0.38 for NRS at rest and 0.62 for NRS at activity for the primary TKA group and 0.11 for NRS at rest and 0.28 for NRS at activity for the revision TKA group.

**Table 3:** Pain, physical performances during activity of daily living and active knee range of motion. Values are mean (SD) unless otherwise stated

	Primary TKA (n: 166)				Revision TKA (n: 51)			
	Before	After	Change	95% CI	Before	After	Change	95% CI
NRS at rest (median, min-max)	0.0 (0–8)	0.0 (0–4)	NA *	NA	0.0 (0–7)	0.0 (0–7)	NA	NA
NRS during activity	5.4 (2.9)	3.7 (2.6)	1.7* (2.6)	[1.3; 2.1]	5.4 (2.9)	4.6 (2.8)	0.8* (1.9)	[0.2; 1.3]
6 min. walking test (m)	421.9 (91.6)	513.1 (97.9)	91.1* (56.6)	[82.4; 100.0]	407.7 (108.9)	496.6 (111.8)	88.8* (60.0)	[72.0; 105.7]
Stair climbing test (sec)	34.8 (18.6)	23.2 (10.3)	11.6* (11.9)	[9.7; 13.4]	38.6 (24.7)	23.1 (9.8)	15.5* (17.4)	[10.6; 20.4]
Active knee extension (^o^)§	3.3 (5.0)	2.2 (3.9)	1.1* (3.0)	[0.6; 1.6]	2.0 (2.9)	1.1 (2.2)	0.9* (2.0)	[0.3; 1.4]
Active knee flexion (^o^)	106.6 (15.7)	113.5 (14.9)	6.9* (5.7)	[6.1; 7.8]	105.4 (15.3)	112.4 (14.0)	7.0* (5.6)	[5.4; 8.5]

*NRS* Numerical Rating Scale. Paired samples t-test were used for all outcomes except for NRS at rest, where Wilcoxon Signed Rank test was applied. *: *P*-value < 0.05. NA: Not applicable. §: Full knee extension = 0^o^

The primary TKA group had significant improvement in the physical performance tests. The mean improvement in the 6 min. walking test was 91 m and the time spent in the stairclimbing test decreased by 11.6 s. The revision TKA group demonstrated similar, significant improvements with an increase in walking distance of 89 m and a decrease in time spent stairclimbing of 15.5 s (Table 3). Effect sizes were 0.96 for the 6 min. walking test and 0.77 for the stairclimbing test for the primary TKA group and 0.81 for the walking test and 0.82 for the stairclimbing test in the revision TKA group.

Both the primary and the revision TKA groups showed similar, significant improvements in knee ROM. For knee extension, a mean increase in ROM of 1.1 and 0.9^o^ were observed. For the knee flexion, a mean increase in ROM of 6.9 and 7.0^o^ in the primary and revision TKA groups, respectively, were observed (Table 3). Effect sizes were 0.40 for knee extension and 0.45 for knee flexion in the primary TKA group and 0.35 for knee extension and 0.64 for knee flexion in the revision TKA group.

## Discussion

This retrospective cohort study analyzed data from consecutive patients with post-surgical complications after primary or revision TKA that received three weeks of intensive, personalized multimodal rehabilitation. In line with the hypothesis, we found that both groups showed significant improvements in self-reported outcomes, pain intensities, and physical performances. Therefore, these results provide initial knowledge regarding exercise types and frequency in patients with post-surgical complications after primary or revision TKA in real-life settings.

The results indicate that patients with postoperative complications can benefit from intensive, multimodal rehabilitation. Both the primary and revision TKA groups had significant improvement in all outcome measures, except pain at rest for the revision TKA group. Effect sizes were interpreted as “small” in NRS at rest for both groups, NRS at activity for revision TKA group, knee extension for both groups, knee flexion in the TKA group, and KOOS Pain and QOL for the revision TKA group, as “medium” in NRS at activity and KOOS Pain, Symptoms and QOL for the primary TKA group, KOOS Symptoms and ADL for the revision TKA group, stair-climbing test in the TKA group and knee flexion in the revision TKA group and as “large” for the KOOS ADL in the TKA group, the 6 min. walking test for both groups and the stair-climbing test in the revision TKA group. When reviewing the results, a tendency towards better improvement from the multimodal rehabilitation program in the primary TKA group compared with the revision TKA group was observed. This tendency could indicate that patients undergoing revision TKA may require a longer period of rehabilitation and maybe different treatment protocols than standard care. Generally, patients with revision TKA are more complicated as they have had the pain longer with a stronger impact on the nervous system (e.g. central sensitization) [33]. This assumption is supported by the literature considering the reported lower level of satisfaction and markedly impaired function after revision TKA [7].

Regarding the primary outcome on the KOOS subscales, both the primary and the revision TKA groups experienced improvement ranging from 9–14 and 7–11 points, respectively. Thus, the improvement in KOOS can be considered clinically relevant [28]. However, threshold values for clinical relevance are still a matter of discussion [34]. Moreover, the timing of follow-up assessment might have an impact on the measured improvements in e.g., KOOS. Concerning the NRS scores, decreases in pain intensities were observed at both rest and during activity. A study by Salaffi et al. proposed that a decrease of 1 point in NRS could be interpreted as “slightly better” and a decrease of 2 points on NRS could be interpreted as “much better” and, therefore, proposed a MCID of 1–2 points on NRS [35]. Applied to the current study’s result, it would mean that the decrease in pain during activity for the primary TKA should be considered clinically relevant and the observed pain decreases during activity for the revision TKA group and at rest for both groups lies below what is considered clinically relevant.

The results of the current study indicates that some patients needs a more intensive and comprehensive approach regarding rehabilitation, than the often used 2–3 exercise sessions pr. week [29, 36, 37] underlining the importance of personalized intervention programs. Clinical significant improvements can be observed after an intensive three-week rehabilitation protocol. Due to the multimodal nature of the rehabilitation program, it is not possible to recommend specific elements of the rehabilitation protocol. Consequently, it is unknown if some exercises were more effective or if the combination of exercises were the most important component of the rehabilitation. Patients undergoing primary or revision TKA often exhibit multiple deficits, such as pains, impaired muscle strength and physical ability, poor balance, lack of ability to coordinate movement and recruit specific muscles and poor alignment during walking [6, 9]. This highlights the need for a thorough rehabilitation protocol when addressing deficits in patients after primary and revision TKA, aiming at treating the multi-factorial mechanisms behind the deficits. Seasonal variation in exercises occurred because of the real-life settings, which means that some patients received aquatic exercises while others did not. A secondary analysis (Additional file 3) revealed no clinical or significant differences in the outcomes between these groups. Since overall magnitude of the exercises was kept constant for all patients, this indicates that time spent exercising seems to be more important than inclusion of aquatic exercises or not.

### Limitations

There are obvious limitations in a retrospective cohort study. Due to exclusion of patients because of no data available from the KOOS questionnaire, a secondary analysis between those patients with and without KOOS scores were performed. This analysis revealed no clinical or significant differences regarding pain intensities and functional performance (Additional file 4) reducing the potential role of selection bias. Due to discharge after three weeks, no long-term follow-up data exists. Therefore, it is unknown if the benefits gained from the intensive rehabilitation persists over time. A review has underlined that it is necessary to keep exercising to maintain the benefits [38]. As a part of the educational sessions, patients are taught the need of further exercising and a specific, personalized exercises program is being draw up by the physiotherapist. Due to the observational nature and clinical approach of the study, there was no control group and therefore, the effect of time cannot be taken into consideration. Thus, compared with a control group, the effect sizes might have differed. However, the study reflects the clinical settings the rehabilitation protocol must comply with and allows for overall advices for patients with post-surgical complications after primary and revision TKA.

## Conclusion

The present retrospective study showed that, patients with post-surgical complications, undergoing three weeks of intensive, personalized multimodal rehabilitation after primary or revision TKA resulted in clinical relevant improvement in self-reported outcomes, significant pain relief, and improved physical performances. The results indicated that a tailored multimodal approach might be useful to obtain improvements in a cohort of patients with post-surgical complications after primary and revision TKA. Further randomized control trials assessing the effects of multimodal rehabilitation after primary or revision TKA are warranted.

## Supplementary information

**Additional file 1.** TIDieR-checklist.

**Additional file 2.** Rehabilitation protocol.

**Additional file 3.** **Table S4** illustrating a secondary analysis.

**Additional file 4.** **Table S5** illustrating a secondary analysis.

## Data Availability

The datasets used and/or analyzed during the current study are available from the corresponding author on reasonable request.

## Abbreviations

ADL: Activities of daily living
BMI: Body mass index
KOOS: Knee injury and osteoarthritis outcome score
MCID: Minimal clinically important difference
NRS: Numerical rating scale
OA: Osteoarthritis
QOL: Quality of life
ROM: Range of motion
SD: Standard deviation
TKA: Total knee arthroplasty
