# Supplementary material for: Intensive, personalized multimodal rehabilitation in patients with primary or revision total knee arthroplasty: a retrospective cohort study
Source: BMC Sports Sci Med Rehabil. 2020 Jan 10;12:5. doi: 10.1186/s13102-020-0157-1 (PMC6954561; doi:10.1186/s13102-020-0157-1)
Supplement: Supplementary file 2 — Additional file 2. Rehabilitation protocol. [file 13102_2020_157_MOESM2_ESM.pdf]

Additional file 2:

Intensive, multimodal rehabilitation protocol during 3 weeks of hospitalization. Due to the pragmatic nature of the study, seasonal variation in exercises occurred. Despite this, the total amount (time) of exercising was kept constant during the entire year.

	Summer program (from May to October)	Winter program (from October to May)
**Week 1**	5 x Group-based exercises, 45 min.<br>1 x Resistance training, 45 min.<br>1 x Instruction in gait retraining, 60 min.<br>1 x Education, 60 min.<br>3 x Aquatic exercises (outdoor), 30 min.	5 x Group-based exercises, 50 min.<br>2 x Resistance training, 45 min.<br>1 x Instruction in gait retraining, 60 min.<br>1 x Education, 60 min.
**Week 2**	5 x Group-based exercises, 45 min.<br>2 x Resistance training, 45 min.<br>2-4* x Various Group-based exercises, 45 min.<br>4 x Aquatic exercises (outdoor), 30 min.	5 x Group-based exercises, 50 min.<br>3 x Resistance training, 45 min.<br>4-6* x Various Group-based exercises, 45 min.
**Week 3**	5 x Group-based exercises, 45 min.<br>2 x Resistance, 45 min.<br>1 x Education, 60 min.<br>2-4* x Various Group-based exercises, 45 min.<br>4 x Aquatic exercises, 30 min.	5 x Group-based exercises, 50 min.<br>3 x Resistance training, 45 min.<br>1 x Education, 60 min.<br>4-6* x Various Group-based exercises, 45 min.

All training was supervised by a physiotherapist.* Number of assigned various team exercises depends on the physical condition and capacity of the individual patients and is decided by the physiotherapist.

Template for the rehabilitation protocol:

Group-based exercises: Consists of exercises targeting balance, coordination, stabilizing the hip and knee joints, flexibility and muscle strength. Focus was on alignment of joint, correct posture during exercises and correct activation of muscles without compensatory movements. Exercises included pelvic lifts, sit-ups, sliding exercises, lunges, rubber band exercises, chair stands, and stair climbing.

Resistance training: Initiated with a warm-up session using cardiovascular equipment, such as stationary bikes, treadmills or slide steppers. Resistance training were performed in strength training equipment, such as leg press, seated knee flexion and extension, hip abductor and adductor as well as exercises targeting core muscles and upper body. 2-3 sets with 15-20 repetitions of each exercises were performed with focus on reaching muscular fatigue during the exercises.

Instruction in gait retraining: Patients were instructed in how practice normal gait pattern and thereby, relearning a normal and functional optimal gait pattern. Instructions were given both with and without use of walking aid, given the functional level of the patients.

Education: These group-based sessions had focus is on pain management, information about surgical procedures and the prosthesis and the relevance of exercises as treatment. Patients were encouraged to ask questions and share with their own experiences within the group. Furthermore, the patients were taught self-mobilization techniques to improve knee range of motion.

Various group-based exercises: These group-based sessions consists of specific exercises, targeting various disabilities. Session could include indoor or outdoor balance, functional and

dynamic stability of joints, Nordic walking, aquatic cardiovascular exercises, mindfulness or neck-and-shoulder exercises.
