# Supplementary material for: Intensive, personalized multimodal rehabilitation in patients with primary or revision total knee arthroplasty: a retrospective cohort study
Source: BMC Sports Sci Med Rehabil. 2020 Jan 10;12:5. doi: 10.1186/s13102-020-0157-1 (PMC6954561; doi:10.1186/s13102-020-0157-1)
Supplement: Supplementary file 3 — Additional file 3. Table S4 illustrating a secondary analysis. [file 13102_2020_157_MOESM3_ESM.pdf]

Additional file 3:

Table S4: Improvements in all outcomes from before to after rehabilitation. Analysis are between groups comparisons based on the inclusion or not of aquatic exercises in the intensive, multimodal rehabilitation program. Values are mean (SD). For simplicity, results are provided as groups of both primary and revision total knee arthroplasty (TKA) patients, since there were no differences between primary and revision TKA.

	Improvement in "received aquatic exercises" group (n: 119)	Improvement in "did not receive aquatic exercises" group (n: 98)	Difference	95% CI
KOOS Pain	9.0 (14.2)	9.3 (15.8)	0.3	-4.3 ; 3.8
KOOS Symptom	9.0 (14.2)	9.2 (13.8)	0.2	-3.9 ; 3.6
KOOS ADL	14.2 (13.9)	12.4 (12.9)	2.0	-1.7 ; 5.5
KOOS QOL	8.7 (16.8)	8.3 (15.6)	0.4	-4.0 ; 4.8
NRS at rest	-0.3 (1.0)	-0.4 (1.3)	0.1	-0.2 ; 0.4
NRS during activity	-1.7 (2.6)	-1.2 (2.4)	-0.5	-1.2 ; 0.2
6 min. walking test (m)	89.0 (50.3)	92.5 (65.0)	-3.5	-18.9 ; 12.0
Stair climbing test (sec)	-13.2 (15.8)	-11.7 (9.9)	-1.5	-5.0 ; 2.2
Active knee extension (°)	-1.0 (2.7)	-1.1 (3.0)	0.1	-0.7 ; 0.9
Active knee flexion (°)	7.3 (5.7)	6.5 (5.5)	0.9	-0.7 ; 2.4

KOOS: Knee injury and osteoarthritis outcome score. CI: Confidence interval. ADL: Activities of daily living. QOL: Quality of life. NRS: Numerical Rating Scale. Independent samples t-test was used for all outcomes.
