# Supplementary material for: Intensive, personalized multimodal rehabilitation in patients with primary or revision total knee arthroplasty: a retrospective cohort study
Source: BMC Sports Sci Med Rehabil. 2020 Jan 10;12:5. doi: 10.1186/s13102-020-0157-1 (PMC6954561; doi:10.1186/s13102-020-0157-1)
Supplement: Supplementary file 4 — Additional file 4. Table S5 illustrating a secondary analysis. [file 13102_2020_157_MOESM4_ESM.pdf]

Additional file 4:

Table S5: Improvements in pain intensity and functional performance outcomes from before to after rehabilitation. Analysis are comparisons between groups with and without knee injury and osteoarthritis outcome score (KOOS) scores. Values are mean (SD). For simplicity, results are provided as groups of both primary and revision total knee arthroplasty (TKA) patients, since there were no differences between primary and revision TKA.

	Improvement in "without KOOS scores" group (n: 91)	Improvement in "with KOOS scores" group (n: 217)	Difference	95% CI
NRS at rest	-0.3 (1.0)	-0.3 (1.2)	0.0	-0.3 ; 0.3
NRS during activity	-1.9 (2.2)	-1.5 (2.5)	-0.4	-1.0 ; 0.2
6 min. walking test (m)	96.9 (66.6)	90.6 (57.3)	-6.3	-8.5 ; 21.1
Stair climbing test (sec)	-13.2 (14.4)	-12.5 (13.5)	-0.7	-4.1 ; 2.7

CI: Confidence interval. NRS: Numerical Rating Scale. Independent samples t-test was used for all outcomes.
